# JAK2/STAT3 Pathway Was Associated with the Protective Effects of IL-22 On Aortic Dissection with Acute Lung Injury

**DOI:** 10.1155/2017/1917804

**Published:** 2017-07-30

**Authors:** Wei Ren, Zhiwei Wang, Zhiyong Wu, Zhipeng Hu, Feifeng Dai, Jinxing Chang, Bowen Li, Huagang Liu, Yongle Ruan

**Affiliations:** ^1^Department of Cardiovascular Surgery, Renmin Hospital of Wuhan University, Wuhan 430060, China; ^2^Hubei Key Laboratory of Cardiology, Wuhan 430060, China

## Abstract

Patients with aortic dissection (AD) may present acute lung injury (ALI) that may affect the prognosis. In this study, we aim to investigate the roles and mechanism of IL-22 in the pathogenesis of AD complicated with ALI. Six hundred and twenty-one AD patients were included, and the incidence of ALI and pulmonary CT findings were analyzed. Mouse ALI model was established through AngII, and then IL-22 injection and AG490 were given. The pathological changes, infiltration of inflammatory cells, and expression of STAT3 were determined. For the in vitro experiment, cultivated pulmonary microvascular endothelial cells (PMVECs) were treated by angiotensin II (AngII), followed by treating with IL-22 and/or AG490. The expression and migration of STAT3 was determined. Flow cytometry was carried out to evaluate the apoptosis. IL-22 contributed to the expression of STAT3 in lung tissues and attenuation of ALI. IL-22 obviously inhibited the apoptosis of PMVECs mediated by AngII and downregulated the expression and intranuclear transmission of STAT3. Such phenomenon was completely inhibited upon administration of AG490, an inhibitor of JAK2. Our data showed IL-22 contributed to the inhibition of PMVEC apoptosis mediated by AngII through activating the JAK2/STAT3 signaling pathway, which may attenuate the ALI induced by AngII.

## 1. Introduction

Patients with aortic dissection (AD) may present acute lung injury (ALI), and the treatment outcome is much severe than those with single AD [1, 2]. Our previous data indicated the concentration of serum angiotensin II (AngII) in the AD patients complicated with ALI was higher than that in the normal individuals and those with AD [3]. As previously described, AngII was reported to induce apoptosis in the pulmonary microvascular endothelial cells (PMVECs). This may cause interruption to the pulmonary microvascular endothelial barrier integrity and increase microvascular permeability, which finally results in ALI.

Interleukin-22 (IL-22), a member of IL-10 family, is initially discovered in 2000 by Dumoutier et al. [4]. As a protective factor of inflammation, IL-22 could bind with the receptors at the surface of the endothelial cells and then activate the STAT signaling pathway. Meanwhile, IL-22 is reported to contribute to the expression of antiapoptosis genes and various antibiotic peptides. Furthermore, it plays crucial roles in the pathogenesis of certain autoimmune diseases such as psoriasis, inflammatory bowel disease, and systemic lupus erythematosus [5–7]. Up to now, rare studies have been focused on the roles of IL-22 in the pathogenesis of cardiovascular disease, particularly the vascular endothelial cells [8]. In this study, we aim to investigate the roles of IL-22 in the onset of ALI in mice and the cultivated PMVECs treated by AngII.

## 2. Materials and Methods

### 2.1. Subjects

Six hundred and twenty-one AD patients admitted in our department from March 2008 to March 2015 were included in this study. AD was diagnosed based on the CT angiography of aorta. Besides, those with chronic pulmonary disorders, with a long-term history of hormonal therapy or medication of anti-inflammatory agents, were also excluded. The diagnosis of ALI was based on the PaO_2_/FiO_2_ of ≤300 mmHg. Written informed consent was obtained from each patient. The study protocols were approved by the Ethical Committee of Renmin Hospital of Wuhan University.

### 2.2. Induction of ALI Model in Mice

Male mice (8 weeks old), purchased from HFK Bioscience Co., Ltd (C57BL/6J, Beijing, China), were divided into four groups after the one-week adaptation, including (i) control group, fed on a normal diet; (ii) AngII group, subject to AngII (1 *μ*g/kg per minute, Sigma-Aldrich) for 1 week through minipumping; (iii) AngII + IL-22 group, subject to AngII and 20 *μ*g/kg IL-22 (CYT-173, ProSpec) via peritoneal injection; and (iv) AngII + IL-22 + AG490 group, treated by AngII, IL-22, and AG490 (10 mg/kg, sc-202046, Santa Cruz, CA, USA). One week later, the animals were sacrificed after anesthesia using phenobarbital (50 mg/kg) to obtain the lung tissues.

### 2.3. Cell Culture

Rat PMVECs were purchased from BeNa Culture Collection Co., Ltd. (category number BNCC338210; Peking, China). Cells were cultured in endothelial culture medium (number 1001, Sciencell) containing 5% fetal bovine serum (FBS), 1% endothelial cell growth supplement (number 1052, Sciencell) and 1% penicillin/streptomycin solution (number 0503) in 5% CO_2_ at 37°C. Cells (P2–P4) cultured in ECM medium containing no FBS for 24 were divided into four groups: (i) normal control, cultured in low-serum RPMI 1640 medium containing 2% FBS; (ii) AngII group, cells cultured in low-serum RPMI 1640 medium containing 2% FBS and AngII (1 *μ*M, Sigma-Aldrich, St. Louis, USA); (iii) AngII + IL-22 group, cells cultured in low-serum RPMI 1640 medium containing 2% FBS, AngII (1 *μ*M, Sigma-Aldrich, St. Louis, USA), and IL-22 (20 ng/ml, CYT-173, ProSpec); and (iv) AngII + IL-22 + AG490 group, cultured in low-serum RPMI 1640 medium containing 2% FBS, AngII (1 *μ*M, Sigma-Aldrich, St. Louis, USA), IL-22 (20 ng/ml), and AG490 (10 *μ*M, sc-202046, Santa Cruz). After culturing for 72 hrs, the cells were subject to apoptosis analysis and determination of STAT3 expression, respectively.

### 2.4. Electron Microscope

Electron microscope was performed to observe the structural changes of PMVECs. Briefly, the lung tissues obtained from the cadavers with AAD complicated with ALI were fixed using 2.5% glutaraldehyde, followed by washing with phosphate buffer (0.1 M) for 3 hrs. Afterwards, OSO_4_ (2%) was added and mixed for 2 hrs, followed by embedding in the Epon-Araldite. Finally, the samples were subject to staining by uranyl acetate and lead citrate. The images were observed under an H-7700 transmission electron microscope (Hitachi, Tokyo, Japan) to determine the changes of structural changes of PMVECs.

### 2.5. Histopathological Examination

The mouse lung tissues were fixed and embedded as routinely described. The sections (4 *μ*m) were subjected to HE staining to determine the morphology of lung tissues and immunohistochemistry analysis to determine the expression of CD68 and MPO. The images were observed under a BX51 microscope (Olympus Corporation, Tokyo, Japan). The immunohistochemistry images were observed using IPP6.0 software. After antigen retrieval, STAT3 (1: 200, category number ab68153, Abcam) was added and incubated at 4°C overnight. STAT3 was labeled with Cy3 (category number BA1032, Boster Co., Ltd., Wuhan, China) and incubated at 37°C. The expression and localization of the STAT3 was observed using an Eclipse 80i fluorescence microscope (Nikon, Tokyo, Japan).

### 2.6. Western Blotting

The total protein was extracted from the mouse lung tissues and cultured PMVECs. Protein content was evaluated using the BCA commercial kit (Beyotime Biotechnology, Jiangsu, China). The transferred membrane was blocked with 10% skimmed milk for 1 h at room temperature, and then the blocked membrane was incubated with the primary antibody against STAT3 (1 : 1000; Abcam) and *β*-actin (1 : 500; Santa Cruz Biotechnology) overnight at 4°C, respectively. After incubation with the horseradish peroxidase-conjugated secondary antibody (1 : 5000; Zhong Shan-Golden Bridge Biological Technology Company, Peking, China) for 1 h at room temperature, the immunoblotting signals were visualized using a Western luminescent detection kit (Vigorous Biotechnology, Beijing, China).

### 2.7. Flow Cytometry

Cell apoptosis was determined using flow cytometry after annexin V/PI staining. The results were analyzed using Expo32 ADC analysis software.

### 2.8. Statistical Analysis

Quantitative data was presented as the mean ± standard error of mean. Statistical differences were analyzed using Student's *t*-test or one-way ANOVA using Dunnett's test in multiple comparisons. Enumeration data were analyzed using the chi-square test. *P* < 0.05 was considered to be statistically significant.

## 3. Results

### 3.1. Clinical Features of AD Complicated with Lung Injury

**Figure 1:**
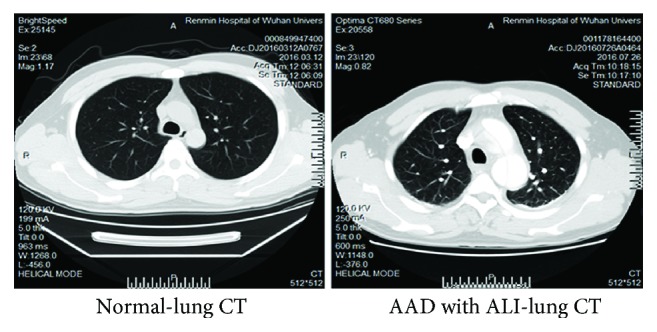
Comparison of pulmonary CT findings in patients with AAD or normal individuals. The pulmonary markings were clear in these patients with no solid shadows or exudation.

In total, 621 cases with AD were included in this study, among which 217 (34.9%) showed concurrent ALI (Table 1). Among the 217 ALI patients, 209 (96.3%) showed AAD within two weeks after onset, while the rest 8 patients (3.7%) showed non-AAD. One hundred and forty cases showed Stanford A type dissection, and 77 showed Stanford B type dissection (Table 2). Compared with the normal individuals, no remarkable differences were noticed in the pulmonary CT findings in those with ALI (Figure 1**)**.

### 3.2. Electron Microscope of the Lung Tissues

**Figure 2:**
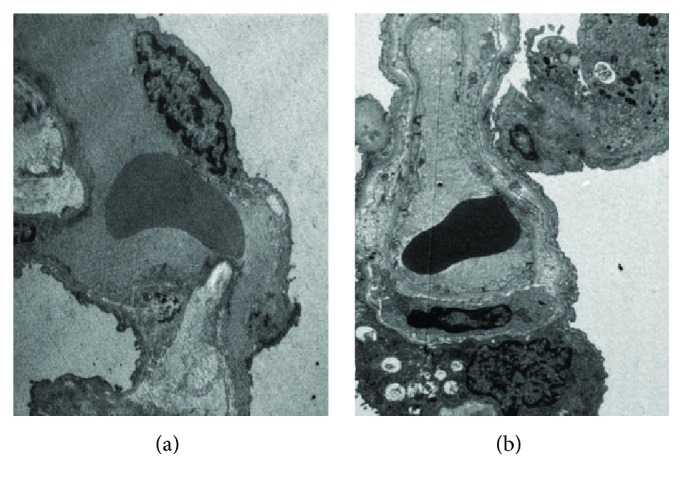
Electron microscope findings in the lung tissues of normal control (a) and patients with AAD complicated with ALI (b). Infiltration of macrophages was observed in the PMVECs in the patients with AAD complicated with ALI, together with karyopyknosis in various forms and chromatin margination. The images were observed under a magnification of 1500x.

As revealed in Figure 2, severe edema was noticed in the lung tissues. Meanwhile, massive accumulation of macrophages was observed in the lung tissues of cadavers with AAD complicated with ALI. Furthermore, proapoptotic lesions were noticed in the PMVECs.

### 3.3. IL-22 Attenuated the ALI Mediated by AngII

**Figure 3:**
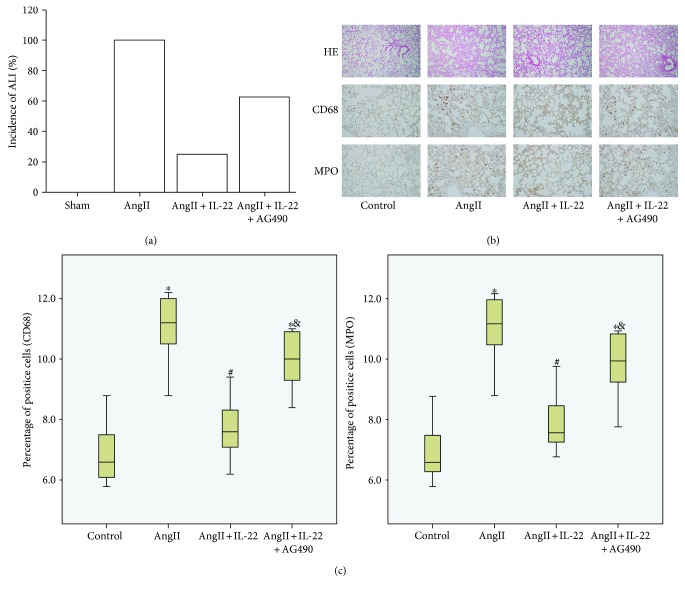
IL-22 induced obvious inhibition of ALI induced by AngII. (a) All mice showed ALI after AngII treatment, and IL-22 could decrease the incidence of ALI. (b) In the AngII group, obvious pulmonary edema was noticed in the lung tissues. (c) Presence of massive infiltration of neutrophils (MPO) and macrophages (CD68). Such phenomenon was completely reversed after IL-22 treatment. AG490 could obviously inhibit the pulmonary protective effects of IL-22. The HE images were observed under a magnification of 100x. The immunohistochemistry images were observed under a magnification of 200x. ^∗^*P* < 0.05, versus the control group; ^#^*P* < 0.05, versus the AngII group; ^&^*P* < 0.05, versus the AngII + IL-22 group.

According to our previous study, serum AngII showed obvious elevation in the ALI patients [3]. On this basis, ALI mouse model was established using AngII through subcutaneous pumping. All the mice in the AngII group showed ALI, and no death was noticed within 1 week (Figure 3(a)). Pathological analysis indicated obvious pulmonary interstitial edema in mice with ALI, together with infiltration of inflammatory cells (Figures 3(b) and 3(c)), whereas the incidence of ALI was obviously decreased after IL-22 treatment. Also, the pulmonary edema and infiltration of inflammatory cells was obviously attenuated after the IL-22 interference. Compared with the AngII + IL-22 group, the protective effects were obviously inhibited in the AngII + IL-22 + AG490 group.

### 3.4. Expression of STAT3 in the Lung Tissues

**Figure 4:**
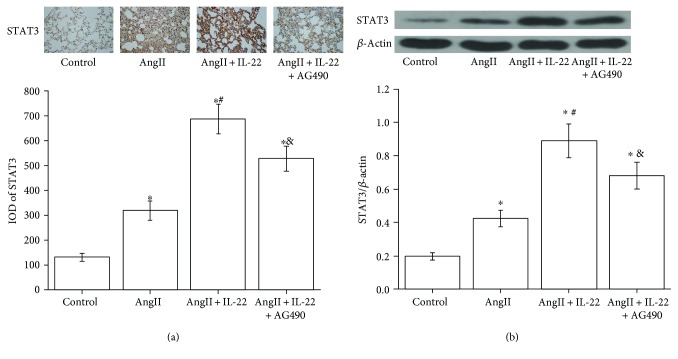
IL-22 contributed to the expression of STAT3 in the mouse lung tissues and was completely inhibited after treating with AG490 as revealed by immunohistochemistry (a) and Western blot analysis (b). ^∗^*P* < 0.05, versus the control group; ^#^*P* < 0.05, versus the AngII group; ^&^*P* < 0.05, versus the AngII + IL-22 group.

The expression of STAT3 in the mouse lung tissues in the AngII + IL-22 group was obviously higher than that of the normal control group and AngII group, respectively, as revealed by the immunohistochemistry analysis and Western blot analysis (Figure 4). In the AngII + IL-22 + AG490 group, the expression of STAT3 in the lung tissues in the mouse obviously decreased compared with the AngII + IL-22 group.

### 3.5. IL-22 Inhibited the Apoptosis of PMVECs Mediated by AngII

**Figure 5:**
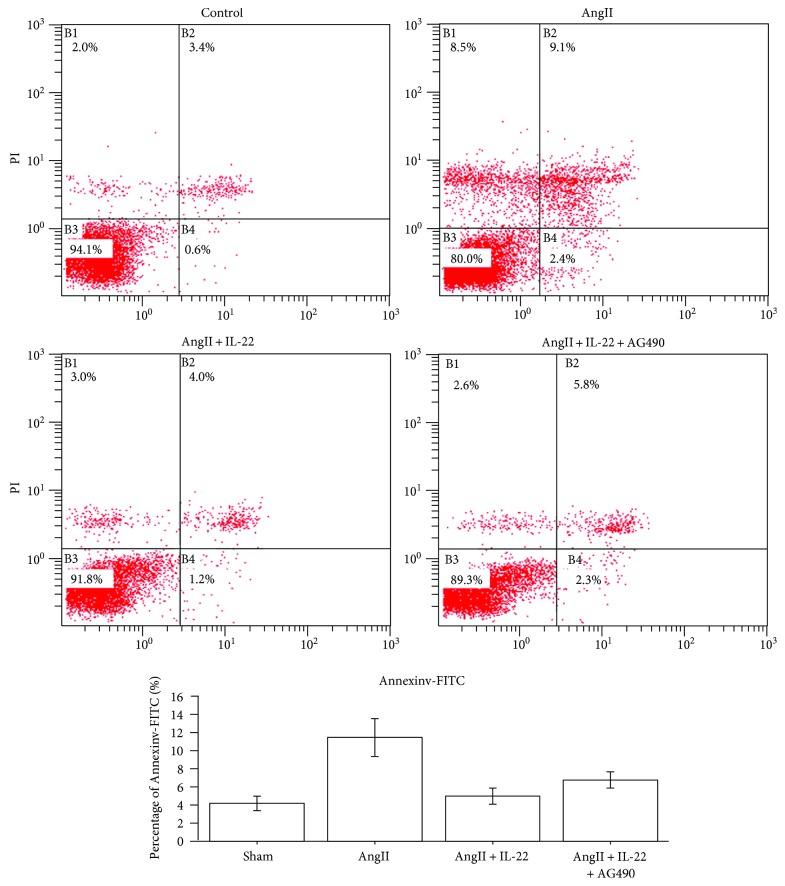
IL-22 inhibited the PMVEC apoptosis induced by AngII. Flow cytometry revealed the apoptosis rate of PMVECs in the IL-22 group was obviously decreased compared with that of the AngII group, whereas the apoptosis rate of PMVECs in the AngII + IL-22 + AG490 group was remarkably increased compared with that of the AngII + IL-22 group.

Our previous data indicated AngII-induced apoptosis of PMVECs was responsible for the onset of ALI [2, 3]. Besides, IL-22 could lead to obvious decrease in the lung injury and attenuate the extent of lung injury. Therefore, we speculated that IL-22 may involve in the attenuation of AngII-mediated apoptosis of PMVECs. Flow cytometry indicated that IL-22 showed obvious antiapoptotic effects on PMVECs (Figure 5).

### 3.6. IL-22 Contributed to the Expression of STAT3 and Intranuclear Transmission

**Figure 6:**
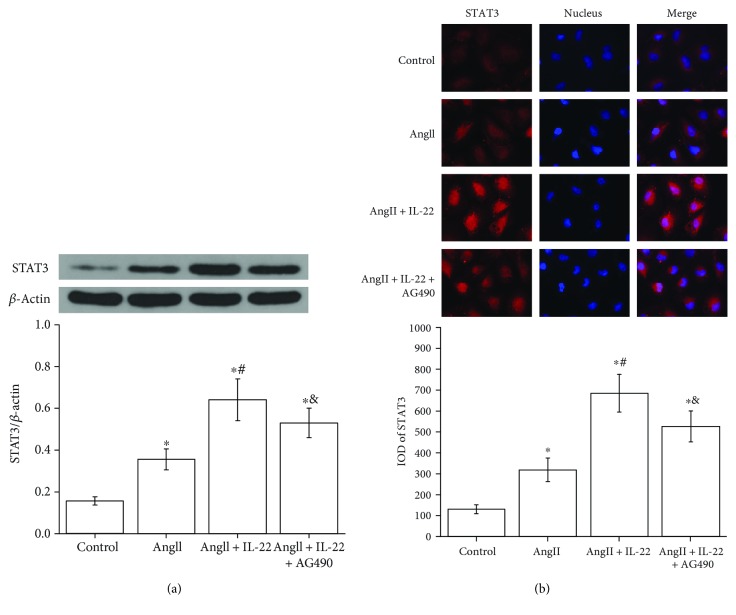
IL-22 contributed to the expression and nuclear transfer of STAT3 in PMVECs. (a) Western blot analysis indicated IL-22 contributed to the expression and nuclear transfer of STAT3; however, such phenomenon was inhibited by AG490. (b) Immunofluorescence assay indicated IL-22 contributed to the expression and nuclear transfer of STAT3, which was attenuated after interference of AG490. ^∗^*P* < 0.05, versus the control group; ^#^*P* < 0.05, versus the AngII group; ^&^*P* < 0.05, versus the AngII + IL-22 group.

JAK/STAT signal pathway plays crucial roles in the IL-22-mediated antiapoptosis and inflammation. In this study, Western blot analysis revealed the expression of STAT3 in the PMVECs subject to AngII + IL-22 was obviously upregulated compared with that of the AngII group (Figure 6(a)). Immunofluorescence analysis revealed the expression of STAT3 in the PMVECs after IL-22 interference was obviously increased and the intranuclear accumulation of STAT3 was enhanced, whereas such phenomenon was completely inhibited after the interference of AG490 (Figure 6(b)).

## 4. Discussion

AD, a severe condition causing great threats to the public health, may trigger multiple organ disorders and systemic inflammation [9–11]. Our previous data indicated the serum AngII increased in those with AAD complicated with ALI [2, 3]. In this study, we aim to investigate the roles of IL-22 in the onset of acute lung injury in mice and the cultivated PMVECs treated by AngII. Our results indicated IL-22 played a crucial role in inhibiting the apoptosis of PMVECs, which could attenuate the ALI induced by AngII.

The roles of AngII in the ALI were mainly featured by inducing systemic inflammation and increase of vascular leakage [12, 13]. PMVECs have been considered as an important target of AngII [14, 15]. AngII could upregulate the expression of cell adhesion molecule and contribute to the chemotaxis and adhesion of neutrophils and monocytes into PMVECs, as well as the accumulation of inflammatory cells. Meanwhile, it could bind the AT1 receptor to activate the transcription of various factors (e.g., NF-*κ*B) and modulate the expression of various inflammatory genes, interleukins, and chemotactic factors [16–18]. Meanwhile, AngII was reported to contribute to the formation of interspace of PMVECs and trigger the increased permeability of pulmonary capillary [19]. Furthermore, it could downregulate the expression of aquaporin 1, decrease the clearance of alveolar fluid, and result in pulmonary edema [20].

In this study, ALI mouse model was established through pumping of AngII, in which obvious edema was noticed in the lung tissues, together with massive infiltration of neutrophils and macrophages, whereas the ALI was attenuated after IL-22 treatment. As a protective factor, IL-22 has been reported to play protective roles in various cells and animal models, such as ischemia-reperfusion injury in lung and active chronic inflammation in the intestine tracts [21–23]. As a member of IL-10 family, IL-22 could be secreted by cells involved in the inherent and adaptive immunity. The IL-22 receptor was a heterogenous dimer which consisted of IL-22R1 and IL-10R2 subunits. Unlike the IL-10 R2 extensively expressed in the cellular surfaces, the IL-22 R1 was only expressed at the surface of epithelial cells in certain organs such as the skin, gastrointestinal tract, pancreas, liver, and lung [24, 25]. Considering the differences of sources and targets of IL-22, it is reasonable to speculate the presence of cross talk between the immunocytes and nonimmunocytes is somehow mediated by IL-22. However, up to now, studies on IL-22 have been focused on the epithelial cells, with rare studies investigating the roles of IL-22 in the endothelial cells and smooth muscle cells in the cardiovascular system [26, 27].

Electron microscope confirmed the proapoptotic changes in PMVECs in the AAD complicated with lung injury, which indicated the apoptosis of PMVECs involving in the pathogenesis of AAD complicated with ALI. Knowing the inhibitory effects of IL-22 on PMVEC apoptosis mediated by AngII, we speculated that IL-22 may play protective roles in the lung injury through inhibiting the PMVEC apoptosis induced by AngII. For the mechanism, IL-22 may bind with the receptors and act on the target cells through activating the JAK/STAT signal pathways, which subsequently induced the phosphorylation of STAT1, STAT3, and STAT5, respectively. Meanwhile, IL-22 could activate the MAPK signal pathway through inducing the phosphorylation of Erk1/2, JNK, and p38 [28, 29].

After IL-22 interference, the expression of signal transducers and activators of transcriptions was obviously upregulated in the PMVECs, together with intranuclear transmission. Such phenomenon was remarkably inhibited by the AG490, a selective inhibitor of JAK kinase family. As a member of protein family involved in the cellular signal transmission, STAT3 has been reported to be participate in the cell growth, differentiation, and apoptosis [30, 31]. JAK/STAT signal pathway consisted of JAK and STAT involves in various biological processes, among which JAK2/STAT3 is considered as a classical pathway for the transcriptional activation and signal transmission of STAT [32]. The binding of the IL-22 and the receptors triggered the dimerization of the receptors, which makes JAK2 kinase and the coupled receptor approaching and activating with each other. Upon the activation of JAK2, the tyrosin residues on the catalytic sites were phosphorylated, which subsequently recruited the STAT3 protein containing the SH2 domain [33, 34]. Finally, the JAK2 kinase may induce phosphorylation of Tyr705 on the STAT3 that bound with the receptor, and then the activated STAT3 would enter the nucleus in a form of a dimer to bind specifically with the DNA sequences to trigger the expression of downstream target genes such as *cyclin D1*, *c-myc*, *c-Jun*, *bcl*, *bcl-xL*, and *mcl-I*. These genes were reported to modulate the cell cycle and inhibit the cell apoptosis, which may be participated in the protective effects of vascular endothelial barrier function [35–37].

The incidence of AD complicated with ALI is more than 30%, and many patients may present hypoxemia. Such condition may induce extended duration of respirator application and pulmonary infection, which is considered as the major cause for the AD-related mortality. Previously, a prevalence of up to 20% was reported in those complicated with ALI [38]. In this study, IL-22 was reported to significantly attenuate the incidence and severity of AngII-induced ALI in mice. Besides, IL-22 could inhibit the AngII-mediated PMVEC apoptosis through modulating the JAK2/STAT3 signaling pathways. Patients with AD complicated with ALI showed elevation of AngII, together with the increased apoptosis of PMVECs. These indicated that IL-22 could inhibit the PMVECs through the JAK/STAT3 signaling pathway, which then attenuated the lung injury. Such aspect may bring in a potential target for the clinical management of AD complicated with ALI, which contributes to the outcome of patients with AD.

## 5. Conclusion

Our data indicated IL-22 may inhibit the PMVEC apoptosis induced by AngII through JAK2/STAT3 signal pathway. This finding contributes to the understanding on the roles of IL-22 in the endothelial cells. It may provide a new treatment target for the AD complicated with ALI.

## Figures and Tables

**Table 1:** Clinical data of AD patients.

Variable	Overall	ALI	Non-ALI	*P* value
*N* (%)	621 (100%)	217 (34.9%)	404 (65.1%)	
Age, y	50.0 ± 9.3	49 ± 6.8	52.1 ± 11.2	
Male sex	502 (80.8%)	185 (85.3%)	317 (78.5%)	0.0425
Smoking	309 (49.8%)	112 (51.6%)	197 (48.8%)	0.5022
Hypertension	573 (92.3%)	204 (94.0%)	369 (91.3%)	0.2718
Acute	480 (77.3%)	209 (96.3%)	271 (67.1%)	<0.0001

**Table 2:** Type of AD complicated with ALI.

Variable	ALI (217)	Non-ALI (404)	*P* value
Stanford A	140	104	
Surgery	118 (82.3%)	88 (84.6%)	1.0000
Interventional therapy	0	0	
Medical management	22 (15.7%)	16 (15.4%)	1.0000
Stanford B	77	300	
Surgery	6 (7.8%)	13 (4.3%)	0.2415
Interventional therapy	54 (70.1%)	209 (69.7%)	1.0000
Medical management	17 (22.1%)	78 (26%)	0.5571

## Abbreviations

AAD: Acute aortic dissection
ALI: Acute lung injury
AngII: Angiotensin II
PMVECs: Pulmonary microvascular endothelial cells
IL-22: Interleukin-22.

## Additional Points

*Availability of Data and Material*. All the data are available upon request.
