# Corrigendum to “JAK2/STAT3 Pathway Was Associated with the Protective Effects of IL-22 on Aortic Dissection with Acute Lung Injury”

**DOI:** 10.1155/2019/1901626

**Published:** 2019-11-21

**Authors:** Wei Ren, Zhiwei Wang, Zhiyong Wu, Zhipeng Hu, Feifeng Dai, Jinxing Chang, Bowen Li, Huagang Liu, Yongle Ruan

**Affiliations:** ^1^Department of Cardiovascular Surgery, Renmin Hospital of Wuhan University, Wuhan 430060, China; ^2^Hubei Key Laboratory of Cardiology, Wuhan 430060, China

The article titled “JAK2/STAT3 Pathway Was Associated with the Protective Effects of IL-22 on Aortic Dissection with Acute Lung Injury” [1] was found to contain material from Wu et al.'s study [2] and to have missing attributions and errors in citations.

In addition, there was figure duplication between both articles [1, 2] as follows:

- The first image in Figure 3(b) in [1] is the same as the second image in Figure 1(c) in [2]
- The second column in Figure 5 in [1] is the same as the one in Figure 3(b) in [2]

The authors apologize for these errors, which have been corrected in the revised version shown below:

- The Introduction, Materials and Methods, and Results sections have been updated
- Figure 3(b) and 5 have been corrected, and their legends have been updated
- Wu et al. [2] has been cited as reference [39]

## 1. Introduction

Patients with aortic dissection (AD) may present acute lung injury (ALI), and the treatment outcome is much severe than those with single AD [1, 2]. The concentration of serum angiotensin II (AngII) in the patients presenting AD complicated with ALI was higher than that in the normal population [3]. As previously described, AngII was reported to induce apoptosis in the pulmonary microvascular endothelial cells (PMVECs). This may cause interruption to the pulmonary microvascular endothelial barrier integrity and increase microvascular permeability, which finally results in ALI. Interleukin-22 (IL-22) is initially discovered in 2000 by Dumoutier et al. [4]. As a protective factor of inflammation, IL-22 could bind with the receptors at the surface of the endothelial cells and then activate the STAT signaling pathway. Meanwhile, IL-22 is reported to contribute to the expression of antiapoptosis genes and various antibiotic peptides. Furthermore, it plays crucial roles in the pathogenesis of certain autoimmune diseases such as psoriasis, inflammatory bowel disease, and systemic lupus erythematosus [5–7]. Up to now, rare studies have been focused on the roles of IL-22 in the pathogenesis of cardiovascular disease, particularly the vascular endothelial cells [8]. In this study, we aim to investigate the roles of IL-22 in the onset of ALI in mice and the cultivated PMVECs treated by AngII.

## 2. Materials and Methods

### 2.1. Subjects

Six hundred and twenty-one AD patients admitted in our department from March 2008 to March 2015 were included in this study. AD was diagnosed based on the CT angiography of the aorta. Besides, those with chronic pulmonary disorders, with a long-term history of hormonal therapy or medication of anti-inflammatory agents, were also excluded. The diagnosis of ALI was based on the PaO2/FiO2 of ≤300 mmHg. Written informed consent was obtained from each patient. The study protocols were approved by the Ethical Committee of Renmin Hospital of Wuhan University.

### 2.2. Induction of AD Complicated with ALI Model in Mice

Male mice (8 weeks old) were provided by HFK Bioscience Co., Ltd. (C57BL/6J, Beijing, China) and were divided into four groups after the one-week adaptation, including (i) the control group, fed on a normal diet; (ii) the AngII group, subject to AngII (1 *μ*g/kg per minute, Sigma-Aldrich) for 1 week through minipumping; (iii) the AngII+IL-22 group, subject to AngII and 20 *μ*g/kg IL-22 (CYT-173, ProSpec) via peritoneal injection; and (iv) the AngII+IL-22+AG490 group, treated by AngII, IL-22, and AG490 (10 mg/kg, sc-202046, Santa Cruz, CA, USA). One week later, the animals were sacrificed after anesthesia using phenobarbital (50 mg/kg) to obtain the lung tissues.

### 2.3. Cells, Antibodies, and Reagents

PMVECs were obtained from the BeNa Culture Collection (BNCC338210; Peking, China). The endothelial culture medium and the supplements were provided by ScienCell (Category No. 1001 and 1052, ScienCell, USA). Antibodies against STAT3 (Category No. ab68153, Abcam, USA) and *β*-actin (Santa Cruz Biotechnology, USA) were used. The IL-22 was acquired from Pro-Spec (CYT-173, Pro-Spec, USA). AngII was provided by Sigma-Aldrich (Sigma-Aldrich, St. Louis, USA), and the JAK2 inhibitor AG490 was obtained from Santa Cruz (sc-202046, Santa Cruz, USA).

PMVECs were cultured as a control group in low-serum RPMI 1640 supplemented with 2% fetal calf serum; AngII (1 *μ*M, Sigma-Aldrich, St. Louis, USA) was added to the AngII group under the above culture conditions; IL-22 (20 ng/ml, CYT-173, ProSpec, USA) was added on the second conditions as the AngII+IL22 group; AG490 (10 *μ*M, sc-202046, Santa Cruz, USA) was added to the AngII+IL22+AG490 group on the third treatment. All groups were assayed after 72 hrs under the same culture conditions.

### 2.4. Electron Microscope

An electron microscope was performed to observe the structural changes of PMVECs. Briefly, the lung tissues obtained from the cadavers with AAD complicated with ALI were fixed using 2.5% glutaraldehyde, followed by washing with phosphate buffer (0.1 M) for 3 hrs. Afterwards, OSO4 (2%) was added and mixed for 2 hrs, followed by embedding in the Epon-Araldite. Finally, the samples were subject to staining by uranyl acetate and lead citrate. The images were observed under an H-7700 transmission electron microscope (Hitachi, Tokyo, Japan) to determine the changes of structural changes of PMVECs.

### 2.5. Histopathological Examination

The mouse lung tissues were fixed and embedded as routinely described. The sections (4 *μ*m) were subjected to HE staining to determine the morphology of lung tissues and immunohistochemistry analysis to determine the expression of CD68 and MPO.

The images were observed under a BX51 microscope (Olympus Corporation, Tokyo, Japan). The immunohistochemistry images were observed using IPP6.0 software. After antigen retrieval, STAT3 (1 : 200, category number ab68153, Abcam) was added and incubated at 4°C overnight. STAT3 was labeled with Cy3 (category number BA1032, Boster Co., Ltd., Wuhan, China) and incubated at 37°C. The expression and localization of the STAT3 were observed using an Eclipse 80i fluorescence microscope (Nikon, Tokyo, Japan).

### 2.6. Western Blotting

For the Western blot analysis, we firstly extracted the total protein from the pulmonary tissues and cultured PMVECs. The BCA method was performed to determine the concentration of protein. The primary antibodies used in this section included STAT3 (1: 1000) and *β*-actin (1: 500) and were incubated overnight at 4°C. Then the horseradish peroxidase-conjugated secondary antibodies (1 : 5000; Zhong Shan-Golden Bridge Biotech, Peking, China) were added and incubated for 1 hr at room temperature. The relative density of the proteins to *β*-actin was analyzed with the TotalLab TL120 Image software.

### 2.7. Flow Cytometry

Cell apoptosis was determined using flow cytometry after annexin V/PI staining. The results were analyzed using Expo32 ADC analysis software.

### 2.8. Statistical Analysis

Data were displayed in the form of the mean ± standard error of mean (SEM) and analyzed using SPSS 16.0 software. Student's *t*-test was utilized for the analysis of statistical differences among different groups. A chi-square test was utilized for the analysis of enumeration data. A *P* value of less than 0.05 was of statistical significance.

## 3. Results

### 3.1. Clinical Features of AD Complicated with Lung Injury

In total, 621 cases with AD were included in this study, among which 217 (34.9%) showed concurrent ALI (Table 1). Among the 217 ALI patients, 209 (96.3%) showed AAD within two weeks after onset, while the rest 8 patients (3.7%) showed non-AAD. One hundred and forty cases showed Stanford A type dissection, and 77 showed Stanford B type dissection (Table 2). Compared with the normal individuals, no remarkable differences were noticed in the pulmonary CT findings in those with ALI (Figure 1).

### 3.2. Electron Microscope of the Lung Tissues

As revealed in Figure 2, severe edema was noticed in the lung tissues. Meanwhile, massive accumulation of macrophages was observed in the lung tissues of cadavers with AAD complicated with ALI. Furthermore, proapoptotic lesions were noticed in the PMVECs.

### 3.3. IL-22 Ameliorated the Concurrent Situation of AD Complicated with ALI through Inhibiting the Pulmonary Edema and Infiltration of Inflammatory Cells

We induced the ALI by AngII pumping (Figure 3(a)) according to the increase of serum AngII in the patients with concurrent situation of AD complicated with ALI [3]. Such type of ALI was mainly characterized by pulmonary edema and inflammatory cell infiltration (Figures 3(b) and 3(c)) and was obviously remised after IL-22 interference. Therefore, we can speculate that IL-22 inhibited the concurrent situation of AD complicated with ALI.

### 3.4. IL-22 Ameliorated the AD Complicated with ALI by Upregulating the Expression of STAT3

As mentioned above, IL-22 could lead to remission of AD complicated with ALI, but the mechanisms were not well defined. IL-22 downstream components were known to modulate the JAK2/STAT3 pathway; then we determined the STAT3 in each group. Our data showed that IL-22 could induce the increase of STAT3 (Figure 4), which may be associated with the remission of the pathogenesis of AD complicated with ALI.

### 3.5. IL-22 Resulted in Remission of AD Complicated with ALI by Inhibiting the Apoptosis of PMVECs

Endothelial cell damage in the blood-gas barrier was the major cause for the pathogenesis of AD complicated with ALI [2, 3]. In this study, after taking the amelioration of IL-22 on AD complicated with ALI into consideration, we investigated the effects of IL-22 on PMVECs. As shown in Figure 5, IL-22 could obviously inhibit the PMVECs mediated by AngII (Figure 5).

### 3.6. IL-22 Contributed to the Expression of STAT3 and Intranuclear Transmission

The JAK/STAT signal pathway plays crucial roles in the IL-22-mediated antiapoptosis and inflammation. In this study, Western blot analysis revealed the expression of STAT3 in the PMVECs subject to AngII+IL-22 was obviously upregulated compared with that of the AngII group (Figure 6(a)). Immunofluorescence analysis revealed that the expression of STAT3 in the PMVECs after IL-22 interference was obviously increased and the intranuclear accumulation of STAT3 was enhanced, whereas such phenomenon was completely inhibited after the interference of AG490 (Figure 6(b)).

## 4. Discussion

AD, a severe condition causing great threats to the public health, may trigger multiple organ disorders and systemic inflammation [9–11]. Our previous data indicated that serum AngII increased in those with AAD complicated with ALI [2, 3]. In this study, we aim to investigate the roles of IL-22 in the onset of acute lung injury in mice and the cultivated PMVECs treated by AngII. Our results indicated that IL-22 played a crucial role in inhibiting the apoptosis of PMVECs, which could attenuate the ALI induced by AngII.

The roles of AngII in the ALI were mainly featured by inducing systemic inflammation and increase of vascular leakage [12, 13]. PMVECs have been considered an important target of AngII [14, 15]. AngII could upregulate the expression of cell adhesion molecule and contribute to the chemotaxis and adhesion of neutrophils and monocytes into PMVECs, as well as the accumulation of inflammatory cells. Meanwhile, it could bind the AT1 receptor to activate the transcription of various factors (e.g., NF-*κ*B) and modulate the expression of various inflammatory genes, interleukins, and chemotactic factors [16–18]. Meanwhile, AngII was reported to contribute to the formation of the interspace of PMVECs and trigger the increased permeability of pulmonary capillary [19]. Furthermore, it could downregulate the expression of aquaporin 1, decrease the clearance of alveolar fluid, and result in pulmonary edema [20].

In this study, the ALI mouse model was established through pumping of AngII, in which obvious edema was noticed in the lung tissues, together with massive infiltration of neutrophils and macrophages, whereas the ALI was attenuated after IL-22 treatment. As a protective factor, IL-22 has been reported to play protective roles in various cells and animal models, such as ischemia-reperfusion injury in the lung and active chronic inflammation in the intestine tracts [21–23]. As a member of the IL-10 family, IL-22 could be secreted by cells involved in the inherent and adaptive immunity. The IL-22 receptor was a heterogeneous dimer which consisted of IL-22R1 and IL-10R2 subunits. Unlike IL-10 R2 extensively expressed in the cellular surfaces, IL-22 R1 was only expressed at the surface of epithelial cells in certain organs such as the skin, gastrointestinal tract, pancreas, liver, and lung [24, 25]. Considering the differences of sources and targets of IL-22, it is reasonable to speculate that the presence of crosstalk between the immunocytes and nonimmunocytes is somehow mediated by IL-22. However, up to now, studies on IL-22 have been focused on the epithelial cells, with rare studies investigating the roles of IL-22 in the endothelial cells and smooth muscle cells in the cardiovascular system [26, 27].

An electron microscope confirmed the proapoptotic changes in PMVECs in the AAD complicated with lung injury, which indicated the apoptosis of PMVECs involving in the pathogenesis of AAD complicated with ALI. Knowing the inhibitory effects of IL-22 on PMVEC apoptosis mediated by AngII, we speculated that IL-22 may play protective roles in the lung injury through inhibiting the PMVEC apoptosis induced by AngII. For the mechanism, IL-22 may bind with the receptors and act on the target cells through activating the JAK/STAT signal pathways, which subsequently induced the phosphorylation of STAT1, STAT3, and STAT5, respectively. Meanwhile, IL-22 could activate the MAPK signal pathway through inducing the phosphorylation of Erk1/2, JNK, and p38 [28, 29].

After IL-22 interference, the expression of signal transducers and activators of transcriptions was obviously upregulated in the PMVECs, together with intranuclear transmission. Such phenomenon was remarkably inhibited by the AG490, a selective inhibitor of the JAK kinase family. As a member of the protein family involved in the cellular signal transmission, STAT3 has been reported to participate in the cell growth, differentiation, and apoptosis [30, 31]. The JAK/STAT signal pathway which consisted of JAK and STAT is involved in various biological processes, among which JAK2/STAT3 is considered a classical pathway for the transcriptional activation and signal transmission of STAT [32]. The binding of the IL-22 and the receptors triggered the dimerization of the receptors, which makes JAK2 kinase and the coupled receptor approaching and activating with each other. Upon the activation of JAK2, the tyrosine residues on the catalytic sites were phosphorylated, which subsequently recruited the STAT3 protein containing the SH2 domain [33, 34]. Finally, the JAK2 kinase may induce phosphorylation of Tyr705 on the STAT3 that bound with the receptor, and then the activated STAT3 would enter the nucleus in a form of a dimer to bind specifically with the DNA sequences to trigger the expression of downstream target genes such as cyclin D1, c-myc, c-Jun, bcl, bcl-xL, and mcl-I. These genes were reported to modulate the cell cycle and inhibit the cell apoptosis, which may participate in the protective effects of vascular endothelial barrier function [35–37].

The incidence of AD complicated with ALI is more than 30%, and many patients may present hypoxemia. Such condition may induce an extended duration of respirator application and pulmonary infection, which is considered the major cause of the AD-related mortality. Previously, a prevalence of up to 20% was reported in those complicated with ALI [38]. In this study, IL-22 was reported to significantly attenuate the incidence and severity of AngII-induced ALI in mice. Besides, IL-22 could inhibit the AngII-mediated PMVEC apoptosis through modulating the JAK2/STAT3 signaling pathways [39]. Patients with AD complicated with ALI showed elevation of AngII, together with the increased apoptosis of PMVECs. These indicated that IL-22 could inhibit the PMVECs through the JAK/STAT3 signaling pathway, which then attenuated the lung injury. Such aspect may bring in a potential target for the clinical management of AD complicated with ALI, which contributes to the outcome of patients with AD.

## 5. Conclusion

Our data indicated IL-22 may inhibit the PMVEC apoptosis induced by AngII through the JAK2/STAT3 signal pathway. This finding contributes to the understanding on the roles of IL-22 in the endothelial cells. It may provide a new treatment target for the AD complicated with ALI.

## Figures and Tables

**Figure 1:**
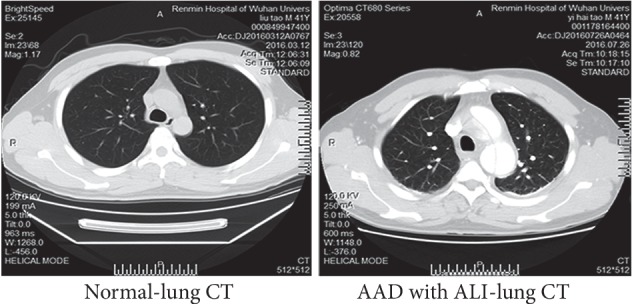
Comparison of pulmonary CT findings in patients with AAD or normal individuals. The pulmonary markings were clear in these patients with no solid shadows or exudation.

**Figure 2:**
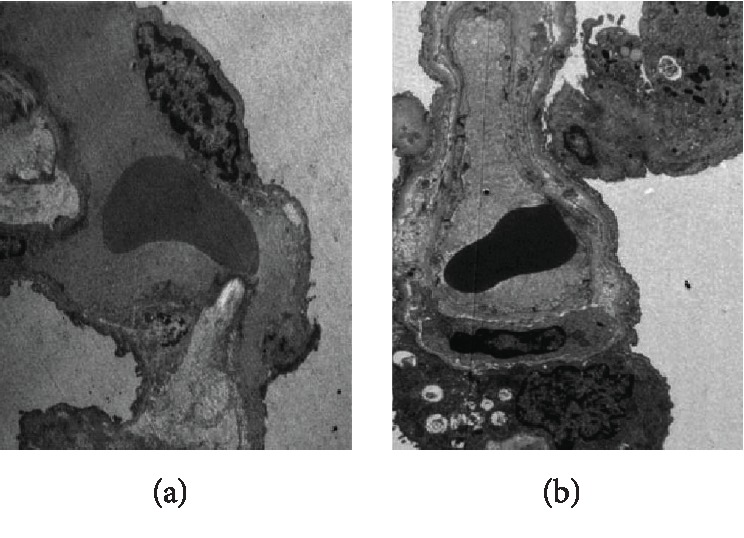
Electron microscope findings in the lung tissues of normal control (a) and patients with AAD complicated with ALI (b). Infiltration of macrophages was observed in the PMVECs in the patients with AAD complicated with ALI, together with karyopyknosis in various forms and chromatin margination. The images were observed under a magnification of 1500x.

**Figure 3:**
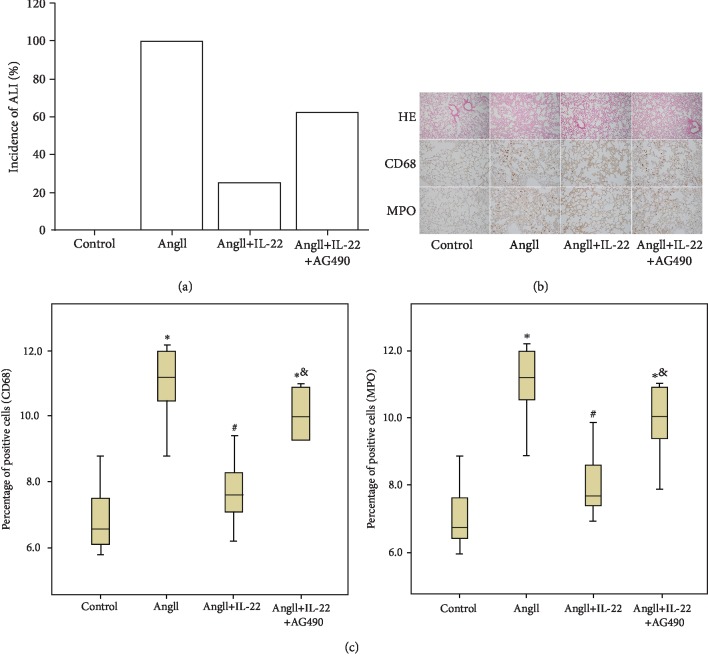
Amelioration of AD complicated with ALI by IL-22. The animal model of AD complicated with ALI was induced by AngII pumping. (a) All mice showed ALI after AngII treatment, and IL-22 could decrease the incidence of ALI. (b) In the AngII group, obvious pulmonary edema was noticed in the lung tissues. (c) There was abundant inflammatory cell infiltration in the model group, including macrophages and neutrophils. Such a process was reversed after IL-22 treatment. The images were observed under a microscope (HE image, 100x; immunohistochemistry images, 200x). ^∗^*P* < 0.05, compared with the control group; ^#^*P* < 0.05, compared with the AngII group; ^&^*P* < 0.05, compared with the AngII+IL-22 group.

**Figure 4:**
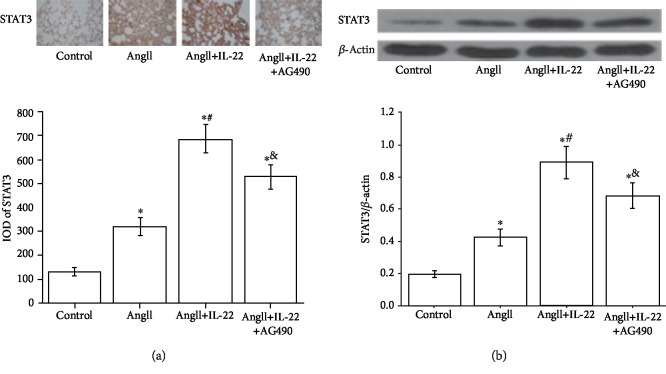
The conditions of AD complicated with ALI were ameliorated by the upregulation of STAT3 mediated by IL-22. After IL-22 interference, the pulmonary edema in the pulmonary tissues showed a remarkable decline compared with that of the AngII group (a). Western blot and immunohistochemisty findings demonstrated the increase of STAT3 mediated by IL-22 in the pulmonary tissues (a, b). ^∗^*P* < 0.05, compared with the control group; ^#^*P* < 0.05, compared with the AngII group; ^&^*P* < 0.05, compared with the AngII+IL-22 group.

**Figure 5:**
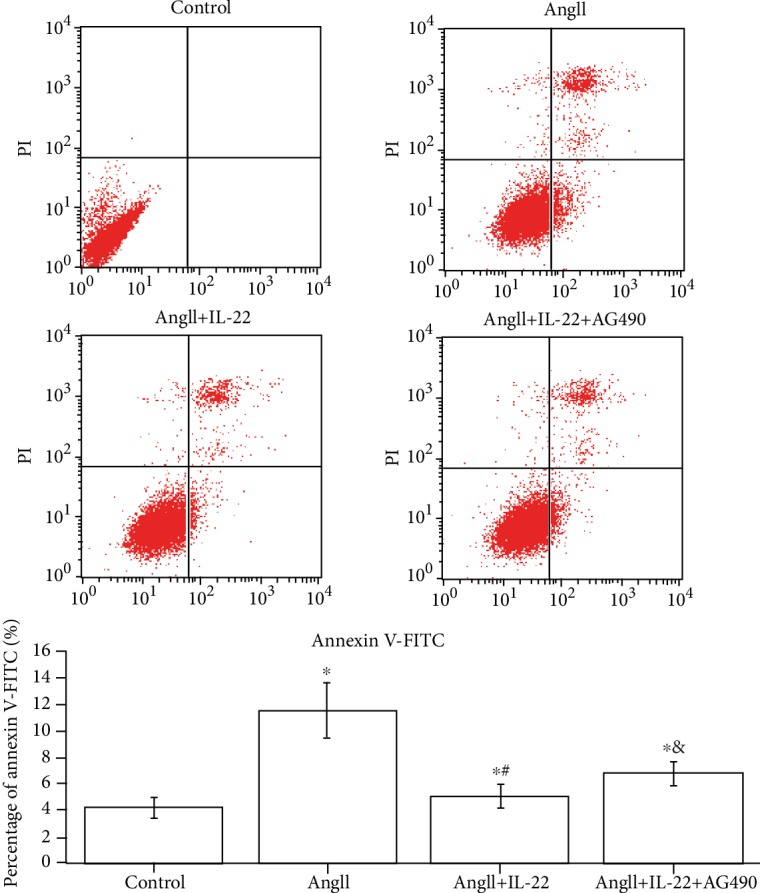
Inhibition of PMVEC apoptosis in AD complicated with ALI mediated by IL-22. Flow cytometry demonstrated a remarkable increase of PMVEC apoptosis in the AngII group. Such phenomenon was reversed after IL-22 treatment. This confirmed that IL-22 inhibited the apoptosis of PMVECs.

**Figure 6:**
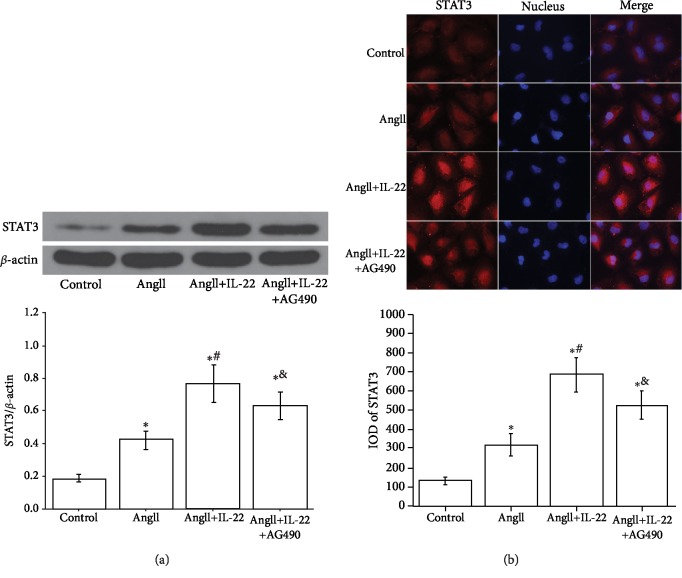
IL-22 contributed to the expression and nuclear transfer of STAT3 in PMVECs. (a) Western blot analysis indicated IL-22 contributed to the expression and nuclear transfer of STAT3; however, such phenomenon was inhibited by AG490. (b) Immunofluorescence assay indicated IL-22 contributed to the expression and nuclear transfer of STAT3, which was attenuated after interference of AG490. ^∗^*P* < 0.05, versus the control group; ^#^*P* < 0.05, versus the AngII group; ^&^*P* < 0.05, versus the AngII+IL-22 group.

**Table 1:** Clinical data of AD patients.

Variable	Overall	ALI	NonALI	*P* value
*N* (%)	621 (100%)	217 (34.9%)	404 (65.1%)	
Age (y)	50.0 ± 9.3	49 ± 6.8	52.1 ± 11.2	
Male sex	502 (80.8%)	185 (85.3%)	317 (78.5%)	0.0425
Smoking	309 (49.8%)	112 (51.6%)	197 (48.8%)	0.5022
Hypertension	573 (92.3%)	204 (94.0%)	369 (91.3%)	0.2718
Acute	480 (77.3%)	209 (96.3%)	271 (67.1%)	<0.0001

**Table 2:** Type of AD complicated with ALI.

Variable	ALI (217)	NonALI (404)	*P* value
Stanford A	140	104	
Surgery	118 (82.3%)	88 (84.6%)	1.0000
Interventional therapy	0	0	
Medical management	22 (15.7%)	16 (15.4%)	1.0000
Stanford B	77	300	
Surgery	6 (7.8%)	13 (4.3%)	0.2415
Interventional therapy	54 (70.1%)	209 (69.7%)	1.0000
Medical management	17 (22.1%)	78 (26%)	0.5571
